# Mast cells and tryptase are linked to itch and disease severity in mycosis fungoides: Results of a pilot study

**DOI:** 10.3389/fimmu.2022.930979

**Published:** 2022-08-10

**Authors:** Dorothea Terhorst-Molawi, Katharina Lohse, Katharina Ginter, Viktoria Puhl, Martin Metz, Man Hu, Marcus Maurer, Sabine Altrichter

**Affiliations:** ^1^ Institute of Allergology, Charité – Universitätsmedizin Berlin, Corporate Member of Freie Universität Berlin and Humboldt-Universität zu Berlin, Berlin, Germany; ^2^ Fraunhofer Institute for Translational Medicine and Pharmacology (ITMP), Allergology and Immunology, Berlin, Germany; ^3^ Institute of Clinical Physiology, Charité – Universitätsmedizin Berlin, Corporate Member of Freie Universität Berlin and Humboldt-Universität zu Berlin, Berlin, Germany; ^4^ Department of Dermatology and Venerology, Kepler University Hospital, Linz, Austria

**Keywords:** mycosis fungoides, cutaneous T-cell lymphoma, mast cell, eosinophil, tryptase, itch (pruritus)

## Abstract

**Introduction:**

In mycosis fungoides (MF), the most common cutaneous T-cell lymphoma, itch is a frequent clinical symptom. Whether mast cells (MCs), eosinophils (Eos) or their mediators play a role in MF-associated itch or disease severity is controversially discussed. Here, we explored the role of MC and Eo numbers in the skin as well as blood levels of their mediators in disease severity and itch.

**Methods:**

In 10 patients with MF and 10 matched control subjects we assessed disease severity, itch, and quality of life impairment using dedicated tools such as the mSWAT, ItchyQoL and DLQI. We analyzed skin biopsies and measured serum levels of tryptase, a mast cell mediator, as well as of the eosinophil products eosinophil cationic protein (ECP) and major basic protein (MBP).

**Results:**

The presence of chronic itch, in four of 10 patients, was associated with significantly higher disease severity (mSwat), larger body surface area affected, and stronger QoL impairment (Itchy-Qol, DLQI). Serum levels of tryptase, but not ECP and MBP, were linked with patient-reported disease severity, body surface area affected, and the presence of itch. Three of the four patients with chronic itch, but none of the six patients without, had tryptase levels above >6µg/l. Numbers of MCs in the papillary dermis were higher in MF skin lesions then in non-lesional skin of MF patients and skin of healthy controls.

**Discussion:**

The MC-mediator tryptase, in MF, is linked to disease activity and impact, most prominently to itch. Our findings call for larger studies that explore the role of MCs, tryptase and other MC mediators as drivers of itch and their role in MF pathogenesis.

## Introduction

Mycosis fungoides (MF) is the most common form of cutaneous T-cell lymphoma (CTCL). MF is caused by the uncontrolled growth of atypical T-cells, usually CD4^+^ T-lymphocytes, and associated inflammation in the epidermis. MF most commonly occurs between 50–70 years of age (1), but can affect people of any age. Neoplastic cells in MF typically express CLA and CCR4, exhibiting the phenotype of skin-homing CD4+ T cells (2). The disease can progress through several stages and is characterized by a skin rash, eczematous lesions and often pruritus (1, 3, 4). In the later stages, intradermal tumors can develop, which may spread into the peripheral blood, lymph nodes and other organs (4). In this stage of the disease, the prognosis is poor with a median survival of less than three years. Monitoring of the disease is crucial in the disease management of these patients. However, no readily assessable biomarkers for disease characterization and monitoring have been identified to date (5).

Itch can be a bothersome symptom and affects more than half of the patients with MF. Itch, in MF, is one of the main drivers of quality of life impairment in these patients (6–8). The exact mechanisms of itch induction in MF lesions are not understood. The degranulation of skin mast cells (MC) and subsequent release of histamine could account for pruritus *via* histamine 1 or 4 receptors. Both receptors are found on sensory skin nerves (9). H1 antihistamines are used for the treatment of pruritus in MF, but this treatment has limited effect (10). In a more recent publication, a role of eosinophils for the itch component in MF was proposed (11), but eosinophils have not been described as common infiltrating cells in early disease stages (12).

Mast cells in the skin, the gastrointestinal tract, and the airways (13, 14) are commonly recognized for their role in IgE-dependent and independent immune responses. MC numbers have been reported to be increased in several hematological and solid cancers (15, 16). Depending on the type of malignancy, MCs have been suggested to have pro-tumorigenic functions, protective effects, or serve as bystanders (17). For example, MCs have been linked to prostate, skin and pancreatic cancer progression (18–20), but have been reported to have protective, anti-tumorigenic effects in breast and lung cancer (21, 22). As for MF, earlier reports have shown that MC numbers are increased in skin lesions and linked to tumor stage and level of invasiveness, suggesting that MCs have a pro-tumorigenic role in MF (10, 15, 16). In support of this notion, MC-deficient mice showed markedly decreased tumor growth in experimental cutaneous lymphoma (4).

The role and relevance of MCs in MF are ill characterized. Human skin MCs produce and release a comprehensive range of biologically active mediators (23). In addition, their activation can drive the recruitment of other proinflammatory cells including basophils, neutrophils and eosinophils (Eos). Changes in Eos numbers in the blood and skin have been linked to MF progression (12, 24, 25). The detection of MC and Eos activation in human skin is challenging, and serological markers have been established as surrogate markers for their activation. Tryptase is only produced by MCs and the most abundant secretory serine protease in MC granules. It is released constantly as well as upon MC activation and is used as a marker for MC numbers as well as MC activation (26). For Eos, blood levels of eosinophilic cationic protein (ECP) and major basic protein (MBP) are used as surrogate markers for cell numbers as well as activation.

Taken together, MC and Eos as well as their mediators appear to be linked to pathogenetic features and the course of MF. To address the gap of knowledge regarding their role on itch and disease severity, we explored the link of MCs, Eos and their activation products with MF disease severity, itch severity and quality of life impairment.

## Materials and methods

### Study participants

In this case-control study, patients with MF were recruited from the Department of Dermatology and Allergy, Charité - Universitätsmedizin Berlin between July 2017 and April 2018. We diagnosed CTCL according to the WHO-EORTC classification for cutaneous lymphomas (27). Patients were required to stop taking local or systemic steroids therapy for the previous two weeks before we performed the tests. We recorded a detailed medical history, took blood samples and biopsies from lesional and non-lesional skin. **Supplementary Figure 1** shows a representative patient photo with characteristic skin lesions. We also recruited ten healthy subjects as a control group who underwent identical procedures (except lesional skin biopsy). Demographic and clinical characteristics of the MF patients and healthy controls are shown in **Table 1**. All 20 participants were skin type II or III according to the classification of Fitzpatrick (28).

**Table 1:** Clinical characteristics of study participants.

	MF patients N=10	Healthy controls N=10	P
**Age [years]**			0.014
Median (IQR),	67.5 (60.5-72)	54.5 (40-63.3)	
Mean ( ± SD)	66.2 ( ± 9.8)	51.6 ( ± 14.2)	
**Sex** M:F	9:1	6:4	0.30
**BMI [kg/m²]**			0.39
Median (IQR),	25.6 (24.1-27.2)	24.5 (22.3-27.0)
Mean ( ± SD)	26.6 ( ± 5.1)	24.5 ( ± 2.9)
**Duration of disease [years]**			–
Median (IQR),	9 (5 – 13.8)	N/A
Mean ( ± SD)	9.8 ( ± 5.7)	
**Stage of disease (ISCL/EORTC, 2007)**			–
**MF IA**	2	N/A
**MF IB**	4	N/A
**MF IIB**	4	N/A
**Palpable Lymph nodes [%]**	10	N/A	–
**Involved Body surface area [%]**			–
Median (IQR),	5.8 (1.8-11)	N/A
Mean ( ± SD)	7.8 ( ± 7.69)	
**mSWAT [points]**			–
Median (IQR),	8 (2.75-16.9)	N/A
Mean ( ± SD)	14.42 ( ± 19.65)	
**Concomitant symptoms:**			–
**Itch [%]**	40	N/A
**Fatigue [%]**	20	N/A
**Insomnia [%]**	20	N/A

MF, Mycosis fungoides; mSWAT, Modified Severity-Weighted Assessment Tool; BMI, Body mass index; N, Number; IQR, Interquartile range; SD, Standard deviation; Continuous variables were compared using Wilcoxon rank test and categorical variables using Chi2 test.

N/A, not applicable.

The Ethics Committee of the Charité - Universitätsmedizin Berlin approved this study (EA4/124/10). The study is registered in the German Clinical Trials Registry (DRKS-ID: DRKS00004277). Presented data are from subcohorts of the trial “ROBERTIS”. Other subcohorts will be presented elsewhere.

### Assessment of clinical parameters

We assessed patients’ global assessment of disease severity *via* a visual analogue scale (VAS) and a Likert scale (0–3). Physicians also assessed disease severity using a Likert scale (0–3). We evaluated the affected body surface area (BSA) using the patient’s palm as a rough reference point for 1%. We determined the skin tumor burden in patients with MF using the modified severity-weighted assessment tool (mSWAT) (29). The mSWAT score is calculated as the sum of affected BSA per body region multiplied by a lesion type-specific weighting factor, with higher values indicating more active disease (30).

Patient-reported outcome measures were applied: All patients assessed itch severity in the last 24 hours, the previous week, and the last month *via* a VAS, as well as itch-related quality of life (QoL) impairment with the help of the ItchyQol questionnaire (31, 32). Patients assessed their skin-related QoL impairment by using the Dermatological Life Quality Index (DLQI) (33).

### Serological analyses

At a central laboratory (Labor Berlin GmbH, Berlin, Germany) the following blood parameters were assessed: differential blood count, total IgE serum levels, tryptase.

Eosinophil-related proteins were analyzed using the following ELISAs: Eosinophilic cationic protein (ECP; MyBioSource MBS700481, San Diego, California, USA) and major basic protein (MBP; MyBioSource MBS9308460). Determinations were performed according to the manufacturer’s instructions.

### Histological analyses

For histological analyses, we took two skin-punch biopsies of 6 mm diameter in the study group (lesional and non-lesional) and one biopsy in the control group (only non-lesional). The non-lesional biopsy was taken approx. 6 cm away from the biopsy of the lesional skin. The proximity of both biopsies assures comparability of MC counts as MC numbers have been shown to vary in different parts of the body (34). After collection, tissue biopsies were immediately fixed in 5% buffered formalin overnight and subsequently embedded in paraffin wax as per routine protocol (Department of Pathology, Charité – Universitätsmedizin Berlin). The wax blocks were cut into 5‐μm sections and stained with Giemsa (Merck KG, Darmstadt, Germany) for histology. **Supplementary Figure 2** shows exemplary stainings.

MCs and Eos were counted by two independent and blinded trained investigators in five or more horizontally adjacent high-power fields (× 400, 0.15 mm2) per skin layer, and mean cell numbers per horizontal layer were calculated. At least three and up to six skin layers were counted per skin sample.

### Statistical Analyses

Datasets were small and hence tests for normal distribution (visually *via* QQ plot, computationally *via* Shapiro-Wilk test) missed normal distribution for most values. For descriptive statistics continuous variables were summarized using median and interquartile range as well as mean and standard deviation. Categorical variables are given as number and percentage. Continuous variables were compared using Wilcoxon rank test and categorical variables using Chi2 test. For sets of matched samples, the Wilcoxon signed rank test and for correlations the Spearman correlation was used. Any missing data was excluded for the respective statistical analysis. A p-value below 0.05 was considered statistically significant. All statistics were performed using RStudio Version 3.6.3 and stored in an encrypted Excel sheet on an internal Charité university hospital server. All analyses were exploratory in nature.

## Results

### Itch is more prevalent in patients with severe MF and drives quality of life impairment

In our analyzed patient cohort, four of 10 MF patients reported regular itching of their lesional skin. MF patients who presented with itch were more severely affected, with significantly higher disease severity scores in the mSwat (p=0.042) and a larger affected body surface area (BSA, p=0.014) compared to patients without itch (**Table 2A**).

**Table 2A:** Clinical characteristics of MF patients with and without itch.

		MF with itch N=4	MF without itch N=6	P
**General**	**Age [years]**			0.28
	Median (IQR)	64.5 (55.8 - 70.2)	68.0 (63.5 - 74.0)	
	Mean ( ± SD)	61.5 ( ± 11.7)	69.3 ( ± 7.7)	
	**Sex: Female**			0.83
	N [%]	1 (25.0%)	0 (0.0%)
	**BMI [kg/m2]**			1.0
	Median (IQR)	25.6 (23.0 – 30.6)	25.6 (24.5 – 26.7)
	Mean ( ± SD)	28.1 ( ± 8.4)	25.6 ( ± 1.3)
	**Duration of disease [years]** Me			0.91
	dian (IQR)	7.0 (5.0 – 11.2)	11.0 (6.0 - 13.8)
	Mean ( ± SD)	9.2 ( ± 6.1)	10.2 ( ± 6.0)
	**Age at onset [years]**			0.48
	Median (IQR)	53.5 (49.0 - 56.8)	57.5 (52.5 - 66.2)
	Mean ( ± SD)	52.2 ( ± 11.5)	59.2 ( ± 9.6)
	**Palpable Lymph nodes**			0.63
	N [%]	4 (100.0%)	4 (66.7%)
	**Stage of disease**			0.43
	MF IA	0 (0.0%)	2 (33.3%)
	MF IB	2 (50.0%)	2 (33.3%)
	MF IIB	2 (50.0%)	2 (33.3%)
**MF specific disease severity**	**mSWAT [points]**			**0.042**
	Median (IQR	**22.1 (10.3 - 40.4)**	**3.5 (2.2 - 8.5)**
	Mean ( ± SD)	**28.6 ( ± 26.2)**	**5.0 ( ± 4.0)**
**Involved body surface area (BSA)**	**BSA [%]**			**0.014**
	Median (IQR	**11.0 (10.4 - 15.0)**	**3.5 (1.2 - 5.4)**
	Mean ( ± SD)	**14.4 ( ± 8.5)**	**3.4 ( ± 2.3)**
**Patient global assessment (PGA)**	**PGA-VAS [0-10 VAS scale]**			**0.025**
	Median (IQR	**6.1 (5.5 - 6.8)**	**0.4 (0.0 - 2.1)**
	Mean ( ± SD)	**6.2 ( ± 1.2)**	**1.5 ( ± 2.1)**
	**PGA-L [Likert scale]**			**0.031**
	Median (IQR)	**1.5 (1.0 - 2.2)**	**0.0 (0.0 - 0.8)**
	Mean ( ± SD)	**1.8 ( ± 1.0)**	**0.3 ( ± 0.5)**
**Quality of life (Qol) assessment**	**PGA-Qol-VAS [0-10 VAS scale]**			**0.013**
	Median (IQR)	**3.5 (2.4 – 5.3)**	**0.2 (0.0 - 0.6)**
	Mean ( ± SD)	**4.2 ( ± 2.7)**	**0.3 ( ± 0.3)**
	**PGA-Qol-L [Likert scale]**			**0.025**
	Median (IQR)	**1.0 (0.8 – 1.5)**	**0.0 (0.0 – 0.0)**
	Mean ( ± SD)	**1.2 ( ± 1.3)**	**0.0 ( ± 0.0)**
	**DLQI [points]**			**0.011**
	Median (IQR)	**5.0 (4.8 – 5.5)**	**0.0 (0.0 – 0.8)**
	Mean ( ± SD)	**5.2 ( ± 1.3)**	**1.2 ( ± 1.3)**
	**ItchyQoL-t [points]**			**0.006**
	Median (IQR)	**2.0 (1.7 – 2.2)**	**1.0 (1.0 – 1.0)**
	Mean ( ± SD)	**2.0 ( ± 0.4)**	**1.0 ( ± 0.0)**

Bold numbers mark significant values.

MF, Mycosis fungoides; mSWAT, Modified Severity-Weighted Assessment Tool; BMI, Body mass index; BSA, Body surface area; PGA, Patient global Assessment; QoL, Quality of Life; DLQI, Dermatology Life Quality Index; N, Number; IQR, Interquartile range; SD, Standard deviation; Continuous variables were compared using Wilcoxon rank test and categorical variables using Chi2 test.

In MF patients with itch, itch-related quality of life was significantly more impaired (Itchy-Qol), but also their overall quality of life impairment as assessed by general patient assessment (VAS, Likert) and DLQI (**Table 2A**), indicating a substantial higher disease burden in MF patients with concomitant itch.

### MF is linked to higher numbers of skin mast cells, but not eosinophils

**Figure 1:**
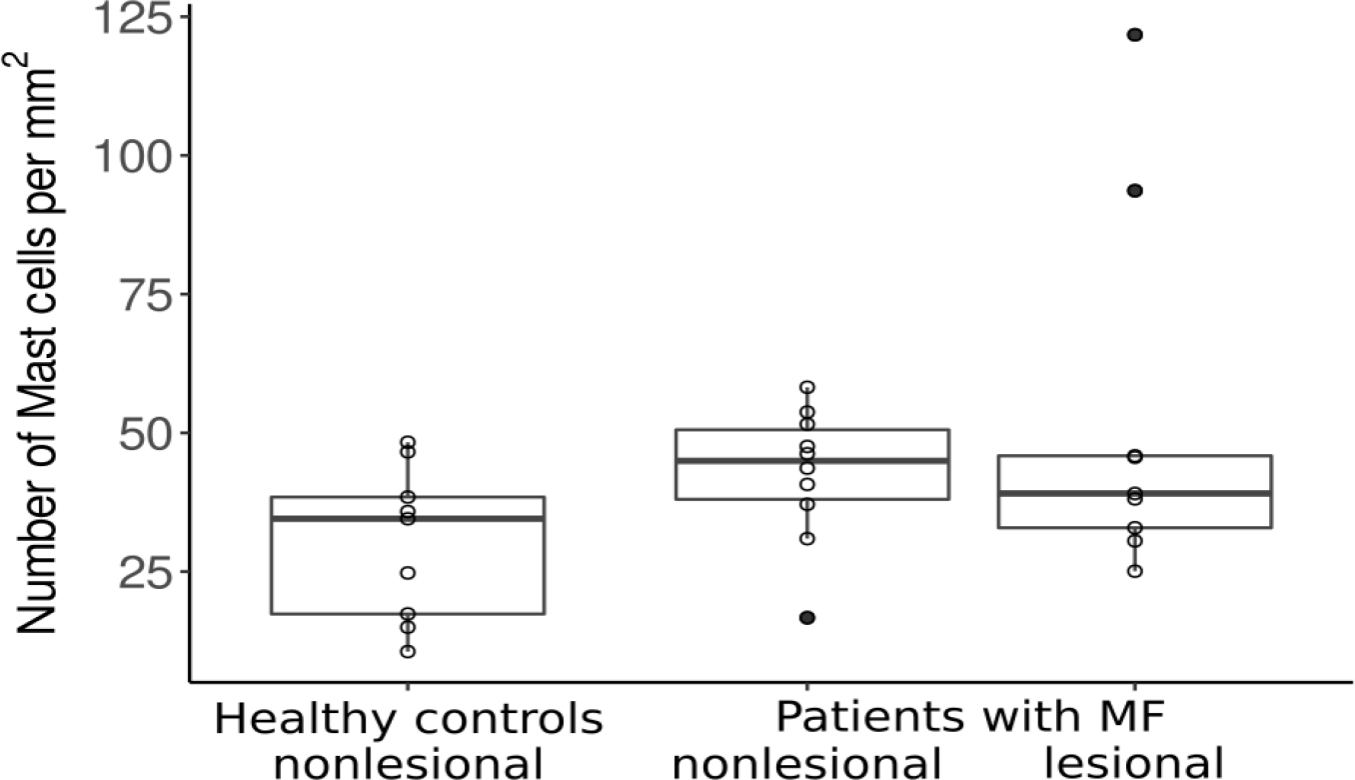
Mast cell counts in the papillary dermis of healthy patients and patients with MF. MF, Mycosis fungoides.

Mast cell (MC) counts in the papillary dermis of MF skin lesions and non-lesional skin were higher than in the skin of healthy controls (**Figure 1**), albeit not statistically significantly. Of note, two MF patients showed a 3-4 fold increase in MC numbers in lesional skin. No differences were seen in MC numbers in the lower parts of the dermis of lesional, non-lesional and healthy skin. Eosinophil numbers were very low in virtually all of skin samples, of MF patients and healthy controls (below 1 cell/HPF); only one MF patient displayed higher eosinophil numbers, i.e. cells/mm² (**Supplementary Figure 2**).

MC and eosinophil numbers did not show significant correlations with any of the assessed clinical markers, like disease severity scores or quality of life scores (see **Supplementary Table 1A**). Of note, the two patients with highly increased MC numbers in lesional skin had lower total skin involvement (BSA of 1-2%) as compared to the other patients. The MF patient with the lowest MC count in lesional skin was among the patients with the highest BSA score (11%), resulting in a trend towards lower MC numbers as a marker for higher skin involvement in MF (but not disease stage).

When looking at the two subgroups of patients with and without itch, we saw comparable MC and eosinophil numbers in both groups (see **Table 2B**).

**Table 2B:** Histological and serological differences of MF patients with and without itch.

		MF with itch	MF without itch	P
Histological parameters	**MC numbers [per mm^2^] (full dermis)**			0.25
	Median (IQR)	18.7 (18.0 - 34.1)	35.1 (29.3 - 52.0)	
	Mean ( ± SD)	28.5 ( ± 18.1)	40.4 ( ± 15.0)	
	**MC numbers [per mm^2^] (papillary dermis)**			0.38
	Median (IQR)	38.1 (31.6 – 41.9)	42.5 (34.4 – 81.7)
	Mean ( ± SD)	36.3 ( ± 10.4)	60.6 ( ± 37.9)
	**Eos numbers [per mm^2^] (full dermis)**			0.88
	Median (IQR)	0.4 (0.2 - 0.4)	0.2 (0.2 - 0.2)
	Mean ( ± SD)	0.3 ( ± 0.3)	0.3 ( ± 0.3)
	**Eos numbers [per mm^2^] (papillary dermis)**			1.0
	Median (IQR)	0.7 (0.3 – 1.1)	0.6 (0.1 - 1.7)
	Mean ( ± SD)	0.8 ( ± 0.8)	0.9 ( ± 1.0)
Serological markers	**Tryptase [µg/l]**			0.16
	Median (IQR)	8.1 (7.0 – 8.5)	5.2 (4.7 - 5.9)
	Mean ( ± SD)	7.4 ( ± 2.1)	5.0 ( ± 1.2)
	**MBP [ng/ml]**			0.35
	Median (IQR)	329.0 (297.6 - 350.0)	428.3 (301.5 - 688.7)
	Mean ( ± SD)	318.7 ( ± 46.4)	694.4 ( ± 777.2)
	**ECP [ng/ml]**			0.17
	Median (IQR)	15.6 (12.2 - 31.9)	11.2 (10.1 - 12.2)
	Mean ( ± SD)	28.5 ( ± 28.8)	14.3 ( ± 10.4)

MC, Mast cell; Eos, Eosinophils; MBP, Major Basic Protein; ECP, Eosinophil Cationic Protein; N, Number; IQR, Interquartile range; SD, Standard deviation; Continuous variables were compared using Wilcoxon rank test; Number of patients: Histological parameters “MF with itch”, N=3, “MF without itch”, N=5 and Serological markers “MF with itch”, N=4, “MF without itch”, N=6.

### In MF, blood levels of tryptase, but not eosinophil mediators, are linked to disease activity, quality of life impairment, and itch

Overall, blood tryptase levels did not differ between MF patients and healthy controls. Also, MBP and ECP serum levels were comparable (see **Supplementary Table 2**).

**Figure 2:**
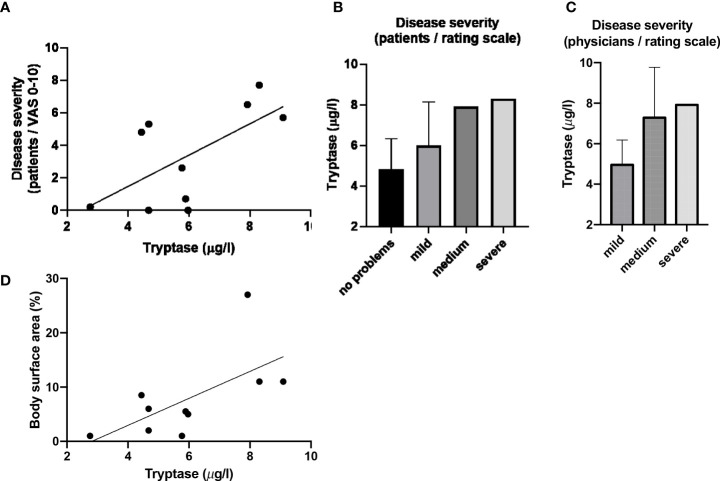
Blood tryptase counts correlate with disease severity and affected body surface area. **(A)** Serum Tryptase levels plottet against severity evaluated by patients using VAS (including depiction of linear correlation). **(B)** Tryptase versus disease severity evaluated by patients using rating scale (values given as mean and SD). **(C)** Tryptase versus disease severity evaluated by physicians using rating scale (values given as mean and SD). **(D)** Serum Tryptase levels plotted against affected body surface area (including depiction of linear correlation).

Blood tryptase was to some extent positively correlated with patient-reported disease severity as assessed by VAS (rS=0.53, p=0.1; **Figure 2**). Patients with more severe disease based on patient or physician global assessment had the highest tryptase levels (**Figure 2**). Tryptase also correlated with affected body surface area (rS=0.63, p=0.05; **Figure 2**). Serum ECP and MBP levels did not correlate with any of the assessed disease severity markers (see **Supplementary Table 1B**).

**Figure 3:**
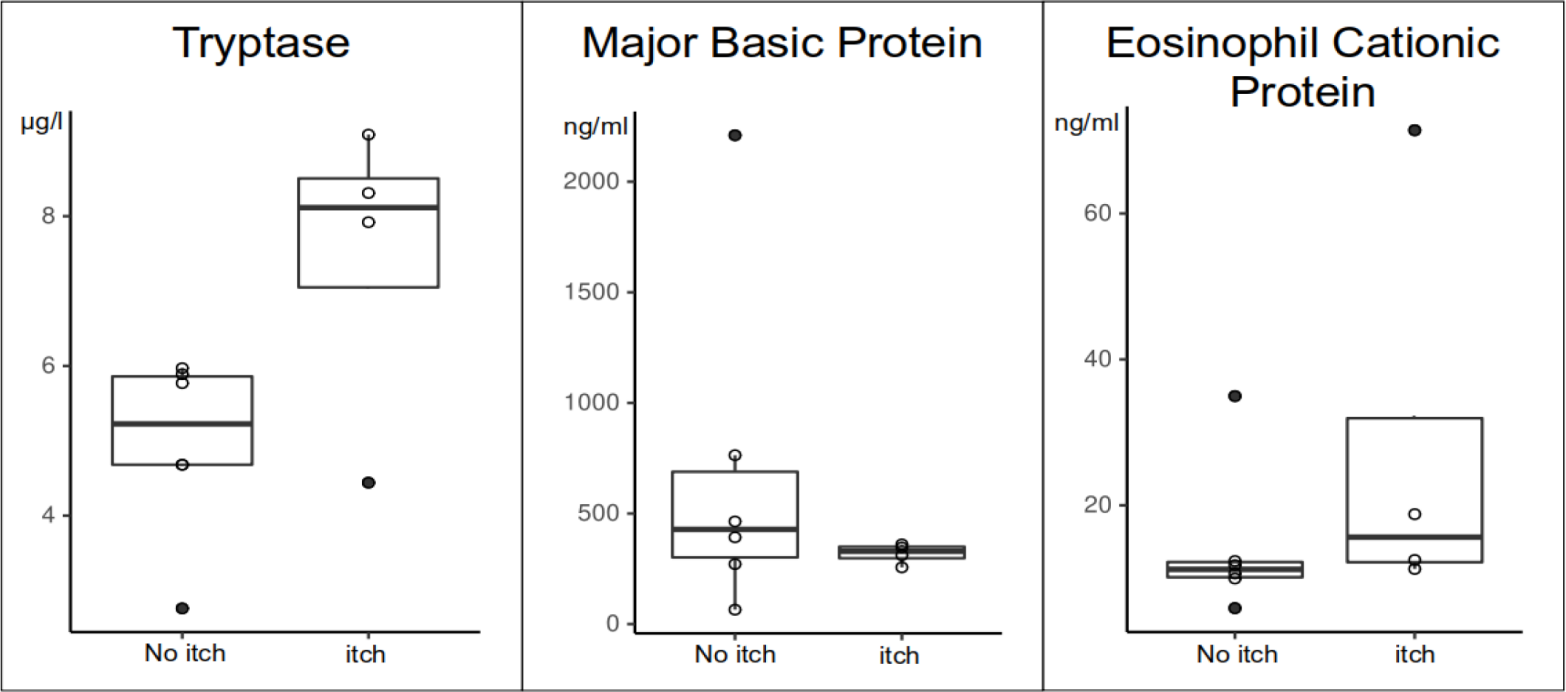
Differences of blood tryptase, MBP and ECP levels in MF patients with itch (itch) and without itch (no itch).

Serum tryptase levels were numerically higher in MF patients with itch (**Table 2B**). Three of the four patients with chronic itch, but none of the six patients without, had tryptase levels above >6µg/l. ECP and MBP levels were not linked to itch (**Table 2B**; **Figure 3**).

## Discussion

Here, for the first time, we aimed to investigate the possible role of MCs, Eos and their mediators in MF by analyzing both skin and blood samples of patients.

Of our ten MF patients, four had chronic pruritus, which is in line with previous reports that itch in MF is frequent, with up to two thirds of patients affected in some studies (35–37). Our findings also confirm that pruritus, in patients with MF, is linked to poor quality of life. Previous MF studies had shown that itch can significantly affect many aspects of quality of life, including sleep (38) and mental wellbeing (39). In line with these reports, we also saw a significantly stronger quality of life impairment in MF patients with pruritus compared to patients without itch in our study. Interestingly, we found a strong and significant difference regarding higher disease severity assessed by BSA and mSWAT in MF patients with itch, irrespective of their disease stage (see **Table 2**). To our knowledge, this has not been reported before, as published reports have shown association with advanced disease stages, but not clinical severity (37, 40).

The underlying pathomechanisms of pruritus in MF and other cutaneous lymphomas remain unknown. MCs have been suspected to play an important role, since elevated numbers had been reported, especially in patients with more advanced stages and pruritus (4, 41). We also reinforce the evidence that MCs are increased in the skin of patients with MF, in our cohort particularly in lesional skin. Of note, the two patients with markedly elevated levels of MCs in the papillary dermis of lesional skin had low BSA involvement (up to 2%) and a lower mSWAT compared to the other patients. This would rather suggest a protective role of MCs in disease control and would be in contrast to prior publications that have shown that increased MC numbers were linked to tumor stage and advanced level of invasiveness, suggesting that MCs have a pro-tumorigenic role in MF (10, 15, 16). These conflicting data should encourage studies that further investigate the role of MCs in MF, since their presence in MF could not only be seen as a pro-tumorigenic, but also as a mechanism of the innate immune system attempting to control the tumor cells. However, since our cohort was very small, no clear conclusion can be drawn for the role on MCs in MF.

A recent study from Japan showed that the number of eosinophils, but not MCs, in the skin of patients with MF were increased in patients with intense itch (11). In our study, neither the number of eosinophils in the skin of MF patients, nor eosinophil-related mediators were elevated or correlated with disease severity, itch, or BSA. Further studies, ideally in larger and more diverse and multicenter patient populations are needed to better characterize the role of eosinophils and eosinophil-related markers in MF.

Previous reports suggest that MCs may be drivers of itch in MF (41), but that histamine is not the key player (3), suggesting that other inflammatory MC mediators such as cytokines, prostaglandin D2, or proteases including tryptase may importantly contribute to itch in MF (42). Tryptase levels in biological fluids have previously been used as an indicator of MC numbers and activation status (43) and increase with age (44) The recently published data from a large population-based cohort hints at lower reference ranges in adults in comparison with the currently applied upper reference limit (44). Tryptase, in our MF patients, was linked to higher disease severity (reported by both patients and physicians). More importantly, serum tryptase levels were higher in the slightly younger MF patients with itch, and three of the four patients with itch had tryptase levels above >6µg/l, which is higher than the average serum tryptase of healthy individuals (45). Indeed, MC tryptase could not only be a surrogate for MC activation, it has been shown to also directly elicit itching in mice through activation of the proteinase-activated receptor-2 (PAR-2), and that the PAR-2 antagonist FSLLRY, inhibits scratching induced by tryptase (46). We, therefore, propose that tryptase could be a significant pruritogen in MF, and further studies should be conducted to investigate this.

Taken together, our results indicate that itch is an important clinical marker in MF which can be easily assessed using simple questionnaires (7, 32) and could be even explored as a tool for disease severity monitoring. Furthermore, our report suggests that MC and their mediator tryptase play a role in itch in MF.

Due to the lack of efficacy of antihistamines (3), alternative treatments for itch in MF should be explored. Currently, patients with severe itch are treated off-label with substances like aprepitant, naloxone, naltrexone, butorphanol, mirtazapine, gabapentin or thalidomide (47), with limited success and often marked side effects. Newer directed therapies (48, 49) aimed at the inhibition or depletion of skin MCs may help treat pruritus, alongside direct lymphoma targeted therapies (47).

The main limitation of our study is the small patient number of only 10, but these patients were thoroughly investigated clinically, serologically, and histologically. Small numbers limit the meaningfulness of statistical tests, and the low patient numbers is likely to be the main reason for some of our comparisons to not show statistical significance. Also, patients were not followed over time to see whether clinical changes were reflected in changes of the assessed histological or serological markers. Also, healthy controls were somewhat younger, but comparable in the assessed cellular and serological marker. However, they should be better matched in regard to sex, age and localization of the biopsies in future studies which should also include patients with more severe and extensive disease.

Nevertheless, our data encourage further exploration of a possible role of MC and their mediators in driving itch and disease severity in MF.

In conclusion, we demonstrated that itch is an important, easily assessable marker in MF and that MCs and the MC mediator tryptase could play an important pathogenic and pruritogenic role. Of course, further analysis, ideally prospective studies in larger cohorts, need to be undertaken to analyze the pathophysiological roles of MCs and tryptase in MF. The investigation of their role as potential therapeutic targets or as markers for disease severity could improve MF patient management.

## Data availability statement

The raw data supporting the conclusions of this article will be made available by the authors, without undue reservation.

## Ethics statement

The studies involving human participants were reviewed and approved by Charité. The patients/participants provided their written informed consent to participate in this study.

## Author contributions

DT-M has collected patient data, was involved in statistical analysis and drafted the manuscript. KL and VP have collected patient data and performed laboratory analyses. KG performed statistical analysis and drafted the manuscript. MMe, MH, and MMa were involved in study planning and proof-reading of the manuscript. SA has planned the study, coordinated the study, collected patient data, performed statistical analysis and drafted the manuscript. All authors were involved in proof-reading of the manuscript and provided input. All authors contributed to the aricle and approved the submitted version.

## Conflict of interest

DT-M has received research funds and was advisor for Celldex, Novartis, Sanofi and Moxie. MMe is or recently was a speaker and/or advisor for AbbVie, Amgen, ArgenX, AstraZeneca, Bayer, Celldex, Celgene, Escient, Galderma, Grünenthal, GSK, Menlo, Novartis, Pfizer, Pharvaris, Roche, Sanofi-Aventis, Third Harmonic Bio. MMa is or recently was a speaker and/or advisor for and/or has received research funding from Allakos, Aralez, Genentech, GSK, Menarini, Merckle Recordati, Moxie, Novartis, Sanofi, MSD, and Uriach. SA has conducted studies for received research funds/was advisor for Allakos, ALK, AstraZeneca, CSL Behring, LeoPharma, Moxie, Novartis, Sanofi, Takeda, Thermofisher. Published results are part of the study ROBERTIS, funded by AstraZeneca.

The remaining authors declare that the research was conducted in the absence of any commercial or financial relationships that could be construed as a potential conflict of interest.

## Publisher’s note

All claims expressed in this article are solely those of the authors and do not necessarily represent those of their affiliated organizations, or those of the publisher, the editors and the reviewers. Any product that may be evaluated in this article, or claim that may be made by its manufacturer, is not guaranteed or endorsed by the publisher.

## Supplementary material

Supplementary Figure 1 Representative photos of a patient with mycosis fungoides. **(A)** An overview of a patient with MF and **(B)** A close-up of the arm, from which both skin biopsies of lesional and non-lesional skin were taken.

Supplementary Figure 2 Mast cell numbers are increased in lesional skin of MF patients. Histological pictures of MC in Giemsa stainings are depicted of healthy control skin **(A, B)**, lesional skin **(C, D)** and non-lesional skin **(E, F)** of a representative MF patient. Pictures **(A, C, E)** are shown as 20 fold magnification, pictures **(B, D, F)** in 40 fold magnification of marked area. Arrows point towards MCs.

Supplementary Figure 3 Postulated mechanism of pruritus induction in MF patients (1).Higher number of mast cells in the papillary dermis (2). Increased levels of tryptase in the skin (3). Tryptase-induced, PAR-2-mediated pruritus

Supplementary Table 1 Correlation of MC numbers in skin with clinical markers of MF. MF, Mycosis fungoides; mSWAT, Modified Severity-Weighted Assessment Tool; BSA, Body surface area; PGA, Patient Global Assessment; QoL, Quality of Life; DLQI, Dermatology Life Quality Index; N, Number; R, Spearman’s R; for correlations the Spearman correlation was used

Supplementary Table 1 Correlation of serological markers with clinical markers of MF. MF, Mycosis fungoides; MBP, Major Basic Protein; ECP, Eosinophil Cationic Protein; mSWAT, Modified Severity-Weighted Assessment Tool; BSA, Body surface area; PGA, Patient Global Assessment; QoL, Quality of Life; DLQI, Dermatology Life Quality Index; N, Number; R, Spearman’s R; for correlations the Spearman correlation was used.

Supplementary Table 2 Comparison of serological and histological markers of MF patients and healthy controls. MF, Mycosis fungoides; MC, Mast cell; Eos, Eosinophils; MBP, Major Basic Protein; ECP, Eosinophil Cationic Protein; N, Number; IQR, Interquartile range; SD, Standard deviation; Continuous variables were compared using Wilcoxon rank test.; Number of patients: Serological markers N=10 and Histological parameters N=9.

## Abbreviations

CTCL, cutaneous T-cell lymphoma; ECP, eosinophil cationic protein; Eos, eosinophil granulocytes; MBP, major basic protein; MC, mast cell; MF, Mycosis fungoides.
