# Supplementary material for: Mast cells and tryptase are linked to itch and disease severity in mycosis fungoides: Results of a pilot study
Source: Front Immunol. 2022 Aug 10;13:930979. doi: 10.3389/fimmu.2022.930979 (PMC9400509; doi:10.3389/fimmu.2022.930979)

Supplementary Figures

Suppl. Figure 1: Representative photos of a patient with mycosis fungoides.

A. An overview of a patient with MF and B. A close-up of the arm, from which both skin biopsies of lesional and non-lesional skin were taken.

A

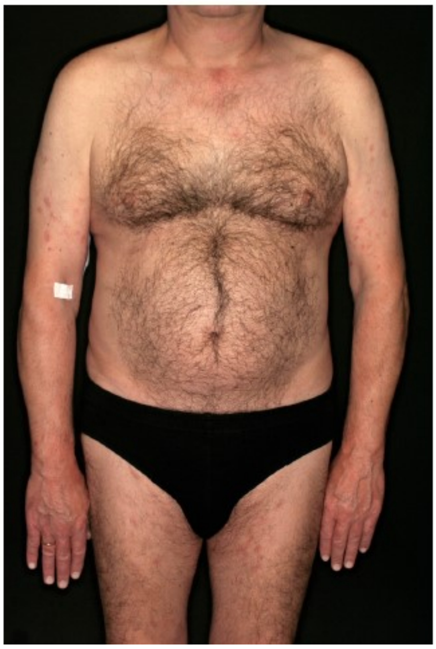

B

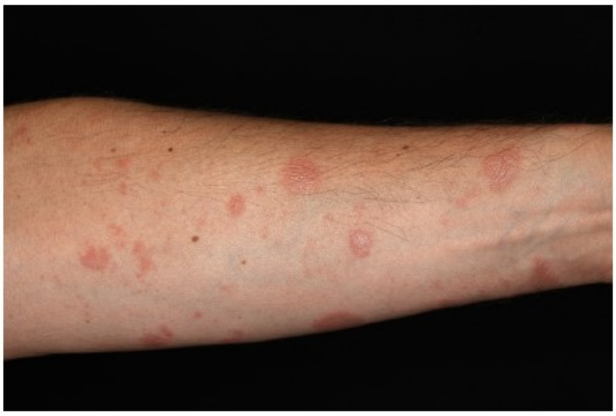

Suppl. Figure 2: Mast cell numbers are increased in lesional skin of MF patients.

Histological pictures of MC in Giemsa stainings are depicted of healthy control skin (A,B), lesional skin (C,D) and non-lesional skin (E,F) of a representative MF patient. Pictures A,C,E are shown as 20 fold magnification, pictures B,D,F in 40 fold magnification of marked area. Arrows point towards MCs.

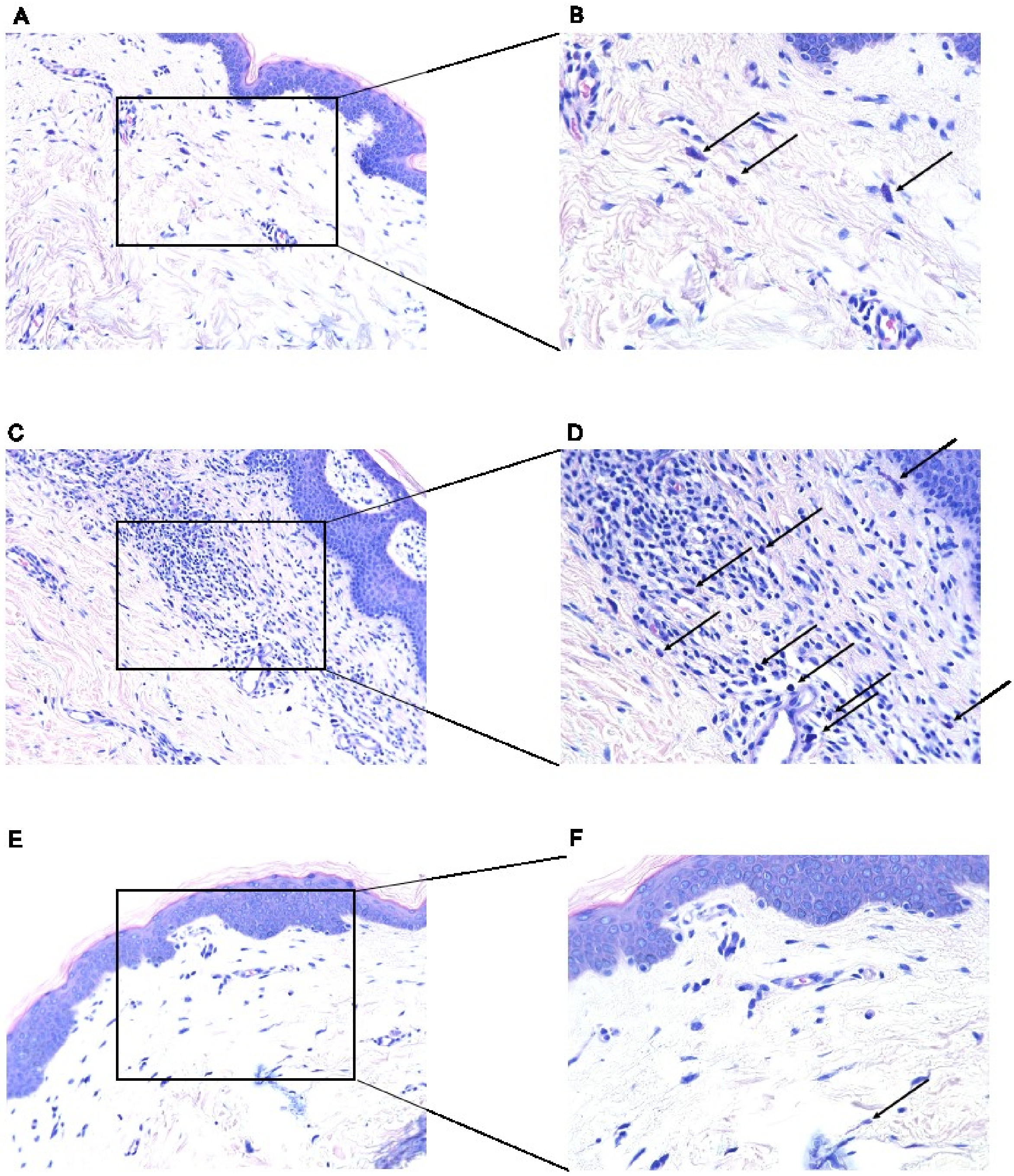

Suppl. Figure 3: Postulated mechanism of pruritus induction in MF patients.

(1) Higher number of mast cells in the papillary dermis
(2) Increased levels of tryptase in the skin
(3) Tryptase-induced, PAR-2-mediated pruritus

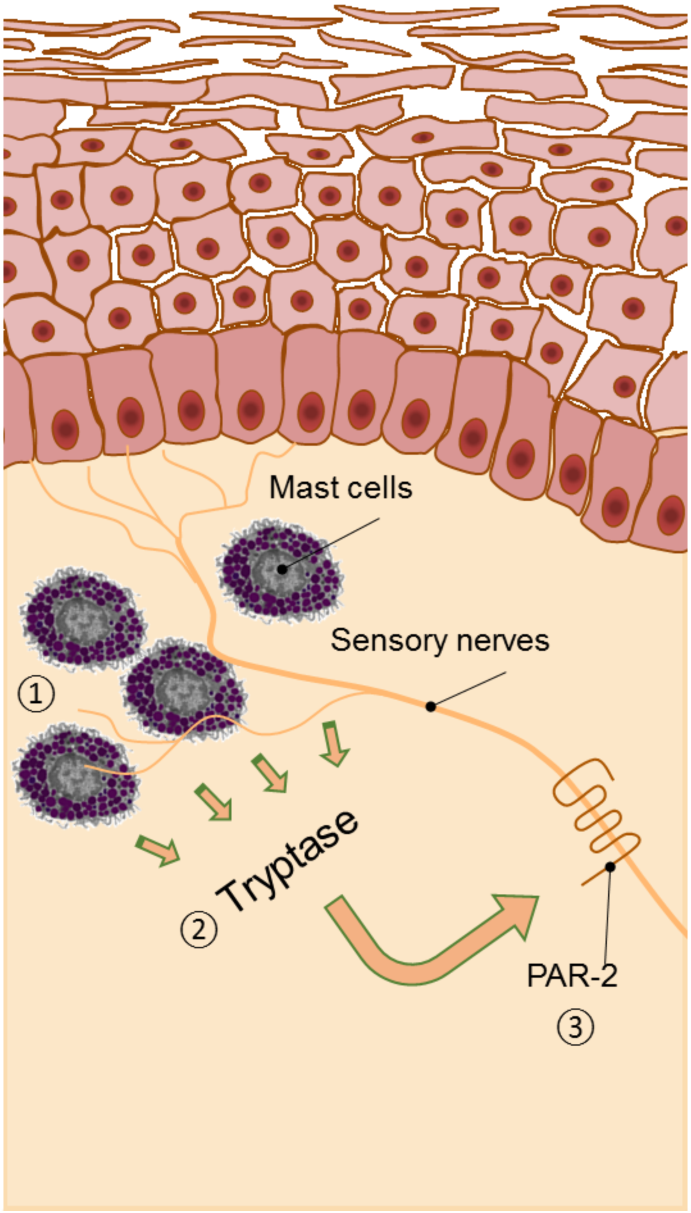

Supplement: Supplementary Figure 1 — Representative photos of a patient with mycosis fungoides. (A) An overview of a patient with MF and (B) A close-up of the arm, from which both skin biopsies of lesional and non-lesional skin were taken. [file Presentation_1.pdf]
