# Supplementary material for: Mast cells and tryptase are linked to itch and disease severity in mycosis fungoides: Results of a pilot study
Source: Front Immunol. 2022 Aug 10;13:930979. doi: 10.3389/fimmu.2022.930979 (PMC9400509; doi:10.3389/fimmu.2022.930979)
Supplement: Supplementary Table 1 — Correlation of MC numbers in skin with clinical markers of MF. MF, Mycosis fungoides; mSWAT, Modified Severity-Weighted Assessment Tool; BSA, Body surface area; PGA, Patient Global Assessment; QoL, Quality of Life; DLQI, Dermatology Life Quality Index; N, Number; R, Spearman’s R; for correlations the Spearman correlation was used [file DataSheet_1.pdf]

Supplementary Tables

Suppl. Table 1a: Correlation of MC numbers in skin with clinical markers of MF.

	**MF**	**Number of MCs of lesional skin, papillary dermis [per mm^2]** N=9		**Number of Eos of lesional skin, papillary dermis [per mm^2]** N=9	
		R	***p***	***R***	***p***
MF specific disease severity	mSwat [points]	-0.46	0.213	0.43	0.251
Involved body surface area	BSA [%]	*-0.61*	*0.081*	0.26	0.491
Patient global assessment	PGA-VAS [0-10 VAS scale]	0.13	0.748	-0.05	0.904
	PGA-L [Likert scale]	0.01	0.982	-0.06	0.88
Itch assessment	Itch within last 24h [0-10 VAS scale]	-0.05	0.897	-0.24	0.53
	Itch within last week [0-10 VAS scale]	-0.03	0.931	-0.18	0.638
	Itch within last month [0-10 VAS scale]	-0.01	0.983	-0.12	0.755
Quality of life assessment	PGA-Qol-VAS [0-10 VAS scale]	0	1	-0.12	0.756
	PGA-Qol-L [Likert scale]	-0.44	0.238	-0.13	0.734
	DLQI [points]	-0.36	0.346	0.24	0.526
	ItchyQoL-total [points]	-0.26	0.504	0.06	0.887

Legend Supplementary Table 1a: MF: Mycosis fungoides; mSWAT: Modified Severity-Weighted Assessment Tool; BSA: Body surface area; PGA: Patient Global Assessment; QoL: Quality of Life; DLQI: Dermatology Life Quality Index; N: Number; R: Spearman's R; for correlations the Spearman correlation was used

Suppl. Table 1b: Correlation of serological markers with clinical markers of MF.

	**MF**	**Tryptase**		**MBP**		**ECP**	
		R	***p***	***R***	***p***	***R***	***p***
MF specific disease severity	mSwat [points]	0.34	0.339	-0.18	0.614	0.18	0.626
Involved body surface area	BSA [%]	*0.63*	*0.051*	-0.23	0.531	0.24	0.497
Patient global assessment	PGA-VAS [0-10 VAS scale]	*0.53*	*0.112*	*-0.36*	*0.3*	*0.15*	*0.688*
	PGA-L [Likert scale]	0.47	0.167	-0.42	0.227	0.12	0.735
Itch assessment	Itch within last 24h [0-10 VAS scale]	0.32	0.367	-0.31	0.379	*0.62*	*0.056*
	Itch within last week [0-10 VAS scale]	0.34	0.342	*-0.37*	*0.294*	*0.57*	*0.084*
	Itch within last month [0-10 VAS scale]	0.38	0.283	-0.44	0.2	0.5	0.142
Quality of life assessment	PGA-Qol-VAS [0-10 VAS scale]	0.28	0.439	-0.46	0.181	0.37	0.287
	PGA-Qol-L [Likert scale]	0.25	0.49	-0.25	0.491	0.2	0.575
	DLQI [points]	0.4	0.257	-0.51	0.128	0.4	0.25
	ItchyQoL-total [points]	0.4	0.247	-0.33	0.345	0.4	0.248

MF: Mycosis fungoides; MBP: Major Basic Protein; ECP: Eosinophil Cationic Protein; mSWAT: Modified Severity-Weighted Assessment Tool; BSA: Body surface area; PGA: Patient Global Assessment; QoL: Quality of Life; DLQI: Dermatology Life Quality Index; N: Number; R: Spearman's R; for correlations the Spearman correlation was used

Suppl. Table 2: Comparison of serological and histological markers of MF patients and healthy controls

		MF patients	Healthy controls	p
Serological markers	**Tryptase [µg/l]** Median (IQR), Mean (±SD)	5.8 (4.7 - 7.4)<br>6.0 (±2.0)	5.2 (4.3 - 6.2)<br>5.2 (±1.3)	0.47
	**ECP [ng/ml]** Median (IQR), Mean (±SD)	12.1 (10.8 - 17.2)<br>20.0 (±19.8)	10.8 (6.1 – 13.4)<br>17.7 (±21.9)	0.39
	**MBP [ng/ml]** Median (IQR), Mean (±SD)	353.5 (281.3 – 446.2)<br>544.1 (±611.5)	317.1 (189.6 – 521.9)<br>381.9 (±245.7)	0.68
	**IgE [ng/ml]** Median (IQR), Mean (±SD)	36.5 (18.4 - 225.8)<br>148.4 (±221.0)	53.4 (27.5 - 203.5)<br>125.1 (±135.3)	0.74
Histological parameters	**MC numbers [per mm^2] (papillary dermis)** Median (IQR), Mean (±SD)	45.0 (38.0 – 50.6)<br>42.6 (±12.1)	34.5 (17.4 - 38.4)<br>30.2 (±13.8)	0.065
	**MC numbers [per mm^2] (full dermis)** Median (IQR), Mean (±SD)	25.7 (22.6 - 29.6)<br>26.3 (±4.5)	25.4 (13.3 – 36.4)<br>26.2 (±15.0)	1.0
	**Eos numbers [per mm^2] (papillary dermis)** Median (IQR), Mean (±SD)	0.0 (0.0 - 0.7)<br>1.4 (±3.5)	0.0 (0.0 - 0.6)<br>0.2 (±0.4)	0.64
	**Eos numbers [per mm^2] (full dermis)** Median (IQR), Mean (±SD)	0.0 (0.0 - 0.5)<br>1.2 (±3.2)	0.0 (0.0 - 0.0)<br>0.0 (±0.1)	0.15

MF: Mycosis fungoides; MC: Mast cell; Eos: Eosinophils; MBP: Major Basic Protein; ECP: Eosinophil Cationic Protein; N: Number; IQR: Interquartile range; SD: Standard deviation; Continuous variables were compared using Wilcoxon rank test. ***; Number of patients: Serological markers N=10 and Histological parameters N=9***
